# Observable Degree Analysis for Multi-Sensor Fusion System

**DOI:** 10.3390/s18124197

**Published:** 2018-11-30

**Authors:** Zhentao Hu, Tianxiang Chen, Quanbo Ge, Hebin Wang

**Affiliations:** 1 College of Computer and Information Engineering, Henan University, Kaifeng 475004, China; hzt@henu.edu.cn; 2 Institute of Systems Science and Control Engineering, School of Automation, Hangzhou Dianzi University, Hangzhou 310018, China; 18767221309@163.com (T.C.); wanghebin@hdu.edu.cn (H.W.)

**Keywords:** multi-sensor network, observable degree analysis, information fusion

## Abstract

Multi-sensor fusion system has many advantages, such as reduce error and improve filtering accuracy. The observability of the system state is an important index to test the convergence accuracy and speed of the designed Kalman filter. In this paper, we evaluate different multi-sensor fusion systems from the perspective of observability. To adjust and optimize the filter performance before filtering, in this paper, we derive the expression form of estimation error covariance of three different fusion methods and discussed both observable degree of fusion center and local filter of fusion step. Based on the ODAEPM, we obtained their discriminant matrix of observable degree and the relationship among different fusion methods is given by mathematical proof. To confirm mathematical conclusion, the simulation analysis is done for multi-sensor CV model. The result demonstrates our theory and verifies the advantage of information fusion system.

## 1. Introduction

Multi-sensor network technology is extensively used in modern life. It has many advantages over single sensor network. However, it faces some new challenges, such as low observability and large data delay [1,2,3]. To some extent, observability can reflect the filtering performance of the system. The low observability caused by complex data collection and translation will deteriorate the estimator performance, and should be given more attention [4,5,6,7]. Thus, it is essential to find a way to guide the multi-sensor netting for improving the estimation performance. The most classic estimator for mobile target tracking is the Kalman filter presented by R. E. Kalman in the 1960s [8]. For the Kalman filtering theory, a basic concept is the observability of state space equation [9]. The observability is used to express the possibility of recovering the initial state by using measurement data and it is related to both state and observation models. Thus, it is important to analyze quantitatively on observability because it can guide the improvement of estimator performance.

For the modern control theory, the observability, which is a qualitative index, can generally be expressed by a variable with two values “0” and “1”. Namely, the result is Boolean value. For zero value, it means that the system is unobservable, which means that the system state is not fully recovered by the measurement. As the quantitative variable, the observable degree is used to measure the observability ability [10]. In [10], an analysis on the observability and observable degree has been given, the kernel is abstracted as follows. Based on the current research work, there are some ways to evaluate the observable degree [11,12,13,14]. For this, observable degree analysis (ODA) has been presented by using estimation error covariance (EEC) of the Kalman filter in [11], which can intuitively express the observable ability of system states or linear combination of state variables. In [12], an evaluation method of observable degree with regard to singular value decomposition has also been given [10]. The observability definition has been further improved by using optimization singular value decomposition method in [13]. Another ODA method, based on pseudo-inverse with the relevant knowledge of least square, is proposed in [14].

The observable degree analysis with estimation performance measure (ODAEPM) proposed by Ma et al., [15] is a great observable degree analysis method, which considers the effect of measurement noise. It reveals the inner relation between the discriminant matrix of observability and the estimation performance of the Kalamn filtering. By synchronously defining the observable degree of state component as local observable degree (LOD) and the observable degree of system as global observable degree (GOD), it establishes the completeness of the observable degree theory.

However, the ODAEPM in [15] only discusses the observable degree problem on single sensor observation. It is necessary to establish the observable degree for multi-sensor network technology. How the fusion methods affect the estimator performance, and the advantage of information fusion should also be verified by the observable degree theory. Further research on observable degree is still needed. Motivated by these, in this paper, following the ODAEPM, we research the observable of different type fusion methods [4,7,16,17], and make the comparison among them. The main contributions of our work are: First, the observable degree discriminant matrix of three different kinds fusion methods, namely the distributed multi-sensor fusion system without feedback, the distributed multi-sensor fusion system with feedback and the centralized multi-sensor sequential fusion system, is derived, mainly based on the estimation error covariance of their fusion process. Second, the mathematical proof of the observable degree relationship between fusion center and local filter is given, along with the relation among different fusion methods, which provides evidence for the advantage of multi-sensor information fusion, and the effect of different fusion methods.

The rest of the paper is organized as follows. First, we review the ODAEPM in Section 2. Then, in Section 3, we introduce the structure of three fusion methods, and, by using ODAEPM, the observable degree analysis is performed on both fusion center and local filter. The comparison of observable degree among different fusion methods is given a mathematical proof in Section 4. In Section 5, the simulation is established to verify our research. Section 6 is the conclusion.

## 2. Review of ODAEPM

### 2.1. Problem Formulation

The associate models of linear time-varying discrete estimation system is considered as [4,18]:

(1) $$\begin{array}{cc} x_{k+1} & =\Phi_{k+1,k}x_{k}+w_{k} \end{array}$$

(2) $$\begin{array}{cc} z_{k} & =H_{k}x_{k}+v_{k} \end{array}$$

where k(k=1,2,…) is time index. $x_{k}\in R^{n}$ is state variable, where *n* is the state dimension. $z_{k}\in R^{m}$ is the observation vector, where *m* is the observation dimension. $\Phi_{k+1,k}\in R^{n\times n}$ is the linear state transition matrix and $H_{k}\in R^{m\times n}$ is the linear observation matrix. $w_{k}\in R^{n}$ and $v_{k}\in R^{m}$ are, respectively, *n*-dimensional Gaussian process noise and *m*-dimensional Gaussian observation noise.

### 2.2. The ODAEPM for Observable Degree Analysis

**Figure 1:**
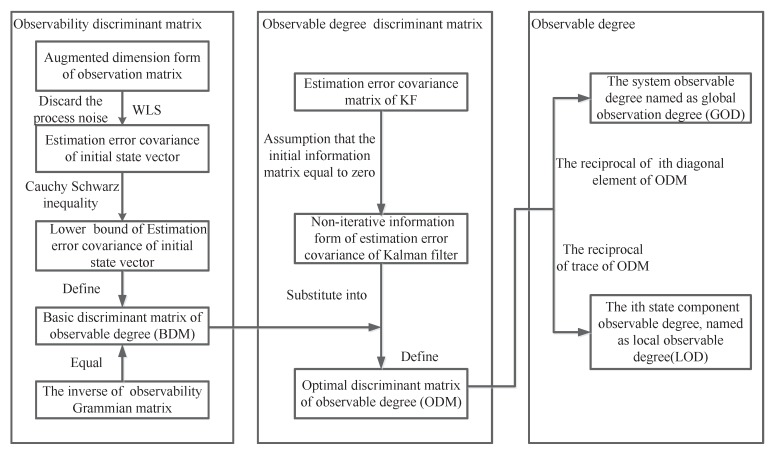
The process of ODAEPM.

ODAEPM [15] is a method dealing with the observable degree analysis for linear time-varying discrete estimation system [15]. The process of ODAEPM is shown in Figure 1.

In Figure 1, we summarize ODAPEM. Here, we need to know that the ODAEPM does not consider the process noise [15]. The ODAEPM names the observable degree of system as global observable degree (GOD):

(3) $$\begin{array}{cc} \eta & =\frac{1}{{Trace(D_{1,k}^{*})}_{}} \end{array}$$

and names the observable degree of state component as local observable degree (LOD):

(4) $$\begin{array}{cc} {\Delta \eta }_{i} & =\frac{1}{{\left(D_{1,k}^{*}\right)}_{i}} \end{array}$$

We define the optimal observability discriminant matrix as:

(5) $$\begin{array}{c} D_{1,k}^{*}=\Phi_{k,1}D_{1,k}{\Phi_{k,1}}^{T} \end{array}$$

Because the non-recursive information form $Y_{k|k}$ of the EEC given the KF related knowledge is as follows:

(6) $$\begin{array}{c} Y_{k|k}={\Phi_{k,0}}^{-T}Y_{0|0}{\Phi_{k,0}}^{-1}+{\Phi_{k,1}}^{-T}{D_{1,k}}^{-1}{\Phi_{k,1}}^{-1} \end{array}$$

Normally, if the system is stable, the system filtering accuracy is independent of the initial value. It may be assumed that $Y_{0|0}\to 0$ , thus Equation (6) can be written as

(7) $$\begin{array}{c} Y_{k|k}\to {\Phi_{k,1}}^{-T}{D_{1,k}}^{-1}{\Phi_{k,1}}^{-1} \end{array}$$

Therefore,

(8) $$\begin{array}{c} P_{k|k}\to \Phi_{k,1}D_{1,k}{\Phi_{k,1}}^{T} \end{array}$$

where $D_{1,k}$ is

(9) $$\begin{array}{c} D_{1,k}=\min\left\{Var\left[x_{1}-{\hat{x}}_{1}\right]\right\}={\left({\vartheta_{1,k}}^{T}R_{1,k}^{-1}\vartheta_{1,k}\right)}^{-1}={\left(\sum_{i=1}^{k}{\Phi_{i,1}}^{T}H_{i}^{T}R_{i}^{-1}H_{i}^{}{\Phi_{i,1}}^{}\right)}^{-1} \end{array}$$

The main contributions of ODAEPM towards observable degree analysis are as follows. First, by constructing observability discriminant matrix by jointly using WLS and Cauchy Schwartz inequality, the uncertainty from the observability effects of observation noise are taken into account. Second, by substituting observability discriminant matrix into the estimation error covariance matrix of KF under the assumption that the initial value of the estimation error covariance matrix tends to be infinite, the relationship between filter estimation performance and observability analysis is clearly established. Finally, by defining the local observable degree (LOD) and global observation degree (GOD), the estimation abilities can be expressed from different scale levels.

## 3. Observable Degree Analysis of Multi-Sensor Observation Network System

Multi-sensor fusion system integrating signals from different sensors has the great advantage of overcoming the uncertainty and limitation under single sensor measurement condition [15,19]. To obtain performance improvements in target tracking problem, multi-sensor observation network becomes an important research field.

### 3.1. Problem Formulation

Distributed multi-sensor track fusion system contains *L* local sensors. The target moving model is

(10) $$x_{k+1}=\Phi_{k+1,k}x_{k}+w_{k}$$

The *i*th local sensor observation model is shown as

(11) $$z_{i,k}=H_{i,k}x_{k}+v_{i,k}$$

where k(k=1,2,…) is time index. The first subscript *i* of matrix **H** and *v* is the sensor index. $x_{k}\in R^{n}$ is state variable, where *n* is the state dimension. $z_{i,k}\in R^{m}$ is *i*th local sensor observation vector, where *m* is the observation dimension. $\Phi_{k+1,k}\in R^{n\times n}$ is the linear state transition matrix and $H_{i,k}\in R^{m\times n}$ is the *i*th local sensor linear observation matrix. $w_{k}\in R^{n}$ and $v_{i,k}\in R^{m}$ are, respectively, *n*-dimensional Gaussian process noise and *m*-dimensional Gaussian observation noise. The covariances of process noise $w_{k}$ and observation noise $v_{i,k}$ are $Q_{k}$ and $R_{i,k}$, respectively.

### 3.2. Introduction of Multi-Sensor Fusion System

There are many forms for multi-sensor fusion system; in this paper, we expound three present major fusion technologies in detail: the distributed multi-sensor fusion without feedback, the distributed multi-sensor fusion with feedback and the centralized multi-sensor sequential fusion.

#### 3.2.1. Distributed Multi-Sensor Fusion without Feedback

**Figure 2:**
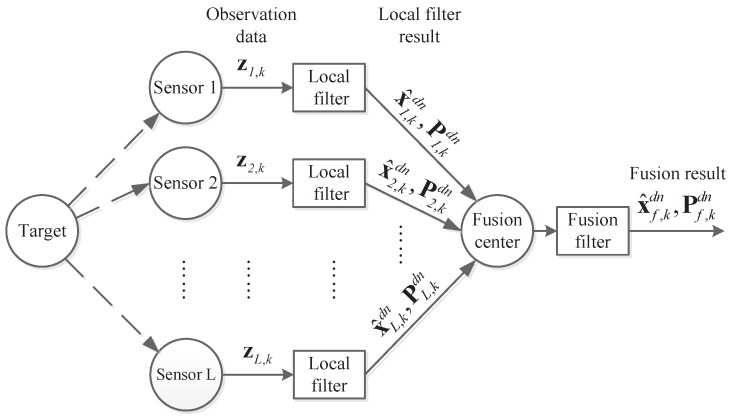
The process of distributed multi-sensor fusion system without feedback.

The structure of distributed multi-sensor fusion without feedback is shown in Figure 2 [4,16,17,18,20].

The distributed multi-sensor fusion system without feedback contains several local sensors. Each sensor performs Kalman filtering on its own observation data, transmitting the state estimation value ${\hat{x}}_{i,k}^{dn}$ and estimation error covariance $P_{i,k}^{dn}$ to the fusion center. After the fusion center gets the filter result form all sensors, the fusion results ${\hat{x}}_{f,k}^{dn}$ and $P_{f,k}^{dn}$ can be calculated by fusion algorithm of distributed multi-sensor fusion system without feedback.

#### 3.2.2. Distributed Multi-Sensor Fusion with Feedback

**Figure 3:**
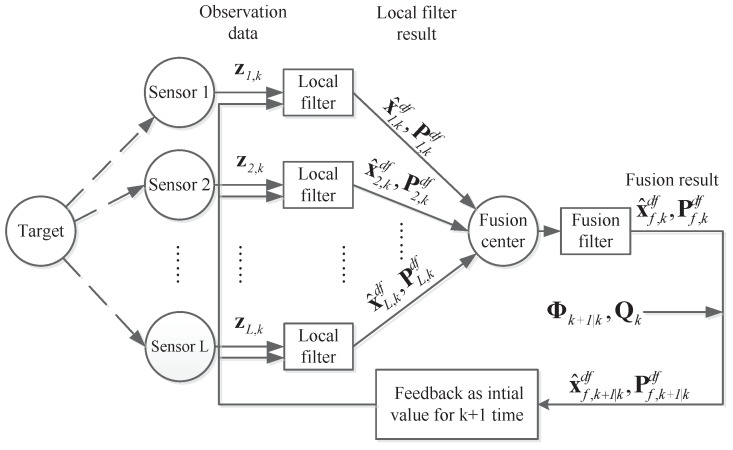
The process of distributed multi-sensor fusion system with feedback.

The structure of distributed multi-sensor fusion with feedback is shown in Figure 3 [4,16,17,18,20].

The form of distributed multi-sensor fusion with feedback is similar to the former fusion technology. The most important difference between these two methods is that the fusion with feedback needs to return the fusion result ${\hat{x}}_{f,k}^{df}$ and $P_{f,k}^{df}$ to the local sensors as their filter initial value at time k+ 1.

#### 3.2.3. Centralized Multi-Sensor Sequential Fusion

**Figure 4:**
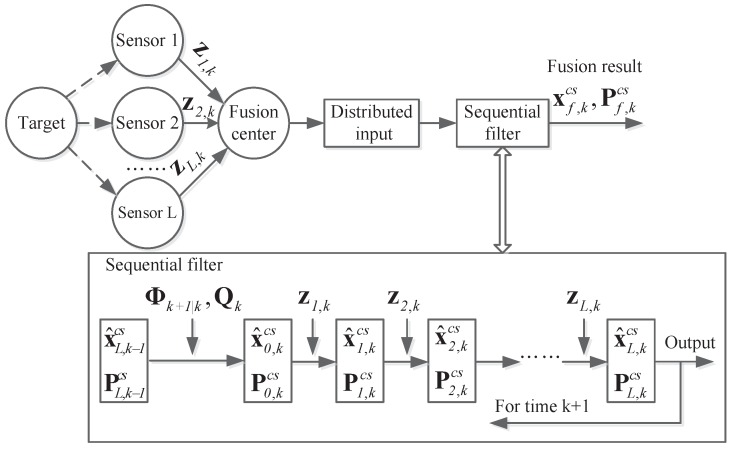
The structure of centralized multi-sensor sequential fusion system.

The structure of centralized multi-sensor sequential fusion is shown in Figure 4 [4,16,17,18,20].

The centralized multi-sensor sequential fusion is different from the other two fusion methods shown in this paper. This fusion technology only contains one sequential fusion filter at fusion center [21,22]. Not only the observation data $z_{i,k}$ but also the observation matrix $H_{i,k}$ are necessary. The messages for same sensor are organized into same group and sequential input to the fusion filter. When each group message is received, the fusion filter will do one sequential filtering and return the result ${\hat{x}}_{i,k}^{cs},P_{i,k}^{cs}$ for next sequential step. When the step index *i* reaches the sensor number *L*, the sequential fusion is finished, and the results ${\hat{x}}_{L,k}^{cs}$ and $P_{L,k}^{cs}$ are the final fusion result for centralized multi-sensor sequential fusion system.

### 3.3. Motivation

Although the ODAEPM proposed by Ma et al., [15] provides a simple way to making observable degree analysis and presents the relationship between the observable degree and estimator accuracy, the problem in [15] is based on the single sensor observation system; the observability theory of multi sensor cooperative target tracking system and estimation of the fusion have not been solved well yet. The relationship of performance between fusion center and local sensors has only been obtained from the mutual independent simulation experiment. There is no mathematical proof yet to verify the advantage of information fusion system. Additionally, how the LOD and GOD of fusion center and local filter influenced by different fusion methods also needs a further study.

### 3.4. Observable Degree Analysis of Multi-Sensor Observation Network System

According to Ma et al., [15], the observable degree discriminant matrix equal to the inverse of non-iterative form of estimation error covariance under the assumption that the initial value of estimation error information matrix equals zero.

#### 3.4.1. Observable Degree Discriminant Matrix of Distributed Multi-Sensor Fusion without Feedback

In distributed multi-sensor fusion system without feedback [23], the local sensor doing Kalman filtering is only based on individual observation data, making it the same as the single sensor observation system. Thus, its local sensors observable degree discriminant matrix at time *k* is the same as the ODAEPM performed on single sensor observation system, shown as

(12) $${\left(D_{1,k}^{*}\right)}_{i}^{dn}={\left[\Phi_{k,1}^{-T}\left(\sum_{j=1}^{k}\Phi_{j,1}^{T}H_{i,j}^{T}R_{i,j}^{-1}H_{i,j}\Phi_{j,1}\right)\Phi_{k,1}^{-1}\right]}^{-1}$$

where $\Phi_{a,b}$ is the state transition matrix between time *a* and time *b*.

Its estimation error covariance matrix of fusion center is:

(13) $${\left(P_{f,k+1}^{dn}\right)}^{-1}={\left(P_{f,k+1|k}^{dn}\right)}^{-1}+\sum_{i=1}^{L}\left[{\left(P_{i,k+1}^{dn}\right)}^{-1}-{\left(P_{i,k+1|k}^{dn}\right)}^{-1}\right]$$

where

(14) $$\begin{array}{cc} (P_{i,k+1|k}^{dn})= & \Phi_{k+1,k}\left(P_{i,k}^{dn}\right)\Phi_{k+1,k}^{T}+Q_{k} \end{array}$$

(15) $$\begin{array}{cc} {\left(P_{i,k+1}^{dn}\right)}^{-1}= & {\left(P_{i,k+1|k}^{dn}\right)}^{-1}+H_{i,k+1}^{T}R_{i,k+1}^{-1}H_{i,k+1} \end{array}$$

Discarding the process noise, Equation (13) can be rewritten as

(16) $${\left(P_{f,k+1}^{dn}\right)}^{-1}=\Phi_{k+1,k}^{-T}{\left(P_{i,k}^{dn}\right)}^{-1}\Phi_{k+1,k}^{-1}+\sum_{i=1}^{L}H_{i,k+1}^{T}R_{i,k+1}^{-1}H_{i,k+1}$$

The fusion center observable degree discriminant matrix ${\left(D_{1,k}^{*}\right)}_{f}^{dn}$ is equal to the inverse of Equation (16) at time *k*, thus changing Equation (16) into the non-iterative form, ${\left(D_{1,k}^{*}\right)}_{f}^{dn}$, will be

(17) $$\begin{array}{cc} {\left(D_{1,k}^{*}\right)}_{f}^{dn} & ={\left[\Phi_{k,k-1}^{-T}{\left(P_{f,k-1}^{dn}\right)}^{-1}\Phi_{k,k-1}^{-1}+\sum_{i=1}^{L}H_{i,k}^{T}R_{i,k}^{-1}H_{i,k}\right]}^{-1}\\ & ={\left[\Phi_{k,k-2}^{-T}{\left(P_{f,k-2}^{dn}\right)}^{-1}\Phi_{k,k-2}^{-1}+\Phi_{k,k-1}^{-T}\sum_{i=1}^{L}H_{i,k-1}^{T}R_{i,k-1}^{-1}H_{i,k-1}\Phi_{k,k-1}^{-1}+\sum_{i=1}^{L}H_{i,k}^{T}R_{i,k}^{-1}H_{i,k}\right]}^{-1}\\ & \vdots \\ & ={\left[\sum_{j=0}^{k-1}\Phi_{k,k-j}^{-T}\sum_{i=1}^{L}H_{i,k-j}^{T}R_{i,k-j}^{-1}H_{i,k-j}\Phi_{k,k-j}^{-1}\right]}^{-1}\\ & ={\left[\Phi_{k,1}^{-T}\sum_{j=1}^{k}\sum_{i=1}^{L}\left(\Phi_{j,1}^{T}H_{i,j}^{T}R_{i,j}^{-1}H_{i,j}\Phi_{j,1}\right)\Phi_{k,1}^{-1}\right]}^{-1} \end{array}$$

#### 3.4.2. Observable Degree Discriminant Matrix of Distributed Multi-Sensor Fusion with Feedback

The recursive formula of estimation error covariance of local sensor in distributed multi-sensor fusion system with feedback [23] is shown as

(18) $${\left(P_{i,k+1}^{df}\right)}^{-1}={\left(P_{i,k+1|k}^{df}\right)}^{-1}+H_{i,k+1}^{T}R_{i,k+1}^{-1}H_{i,k+1}$$

where

(19) $$P_{i,k+1|k}^{df}=\Phi_{k+1,k}P_{f,k}^{df}\Phi_{k+1,k}^{T}+Q_{k}$$

From Equation(18), we need to get the estimation error covariance of fusion center $P_{f,k}^{df}$ before calculating the observable degree of local sensor. According to [19], the result of fusion center estimation error covariance $P_{f,k}^{df}$ is the same as in the fusion system without feedback. Thus, the observable degree discriminant matrix of fusion center ${\left(D_{1,k}^{*}\right)}_{f}^{df}$ is equal to ${\left(D_{1,k}^{*}\right)}_{f}^{dn}$, shown as

(20) $${\left(D_{1,k}^{*}\right)}_{f}^{df}={\left[\Phi_{k,1}^{-T}\sum_{j=1}^{k}\sum_{i=1}^{L}\left(\Phi_{j,1}^{T}H_{i,j}^{T}R_{i,j}^{-1}H_{i,j}\Phi_{j,1}\right)\Phi_{k,1}^{-1}\right]}^{-1}$$

Then, the observable degree discriminant matrix of *i*th local sensor is

(21) $$\begin{array}{cc} {\left(D_{1,k}^{*}\right)}_{i}^{df}= & {\left[\Phi_{k,1}^{-T}\sum_{j=1}^{k-1}\sum_{i=1}^{L}\left(\Phi_{j,1}^{T}H_{i,j}^{T}R_{i,j}^{-1}H_{i,j}\Phi_{j,1}\right)\Phi_{k,1}^{-1}+H_{i,k}^{T}R_{i,k}^{-1}H_{i,k}\right]}^{-1} \end{array}$$

#### 3.4.3. Observable Degree Discriminant Matrix of Centralized Multi-Sensor Sequential Fusion

The centralized multi-sensor sequential fusion method only operates observation data filtering at fusion center [24,25]. The process of estimation error covariance in sequential filtering is

(22) $$\begin{array}{cc} P_{0,k+1}^{cs}= & \Phi_{k+1,k}P_{L,k}^{cs}\Phi_{k+1,k}^{T}+Q_{k} \end{array}$$

(23) $$\begin{array}{cc} {\left(P_{i,k+1}^{cs}\right)}^{-1}= & {\left(P_{i-1,k+1}^{cs}\right)}^{-1}+H_{i,k+1}^{T}R_{i,k+1}^{-1}H_{i,k+1} \end{array}$$

(24) $$\begin{array}{cc} P_{f,k+1}^{cs}= & P_{L,k+1}^{cs} \end{array}$$

The observable degree discriminant matrix of fusion center is

(25) $$\begin{array}{cc} {\left(D_{1,k}^{*}\right)}_{f}^{cs} & ={\left[{\left(P_{L,k}^{cs}\right)}^{-1}\right]}^{-1}={\left[{\left(P_{L-1,k}^{cs}\right)}^{-1}+H_{L,k}^{T}R_{L,k}^{-1}H_{L,k}\right]}^{-1}\\ & ={\left[{\left(P_{0,k+1}^{cs}\right)}^{-1}+\sum_{i=1}^{L}H_{i,k}^{T}R_{i,k}^{-1}H_{i,k}\right]}^{-1}\\ & ={\left[\Phi_{k,k-1}^{-T}{\left(P_{0,k+1}^{cs}\right)}^{-1}\Phi_{k,k-1}^{-1}+\sum_{i=1}^{L}H_{i,k}^{T}R_{i,k}^{-1}H_{i,k}\right]}^{-1}\\ & \vdots \\ & ={\left[\Phi_{k,1}^{-T}\sum_{j=1}^{k}\sum_{i=1}^{L}\left(\Phi_{j,1}^{T}H_{i,j}^{T}R_{i,j}^{-1}H_{i,j}\Phi_{j,1}\right)\Phi_{k,1}^{-1}\right]}^{-1} \end{array}$$

Although centralized multi-sensor sequential fusion does not have a local filter, the result in fusion center is computed step by step with sequential input. Define ${\left(D_{1,k}^{*}\right)}_{i}^{cs}$ as the discriminant matrix of fusion center at *i*th step during time *k*.

(26) $$\begin{array}{cc} {\left(D_{1,k}^{*}\right)}_{i}^{cs}= & {\left[{\left(P_{i,k}^{cs}\right)}^{-1}\right]}^{-1}\\ & ={\left[{\left(P_{0,k+1}^{cs}\right)}^{-1}+\sum_{l=1}^{i}H_{l,k}^{T}R_{l,k}^{-1}H_{l,k}\right]}^{-1}\\ & ={\left[\Phi_{k,1}^{-T}\sum_{j=1}^{k-1}\sum_{i=1}^{L}\left(\Phi_{j,1}^{T}H_{i,j}^{T}R_{i,j}^{-1}H_{i,j}\Phi_{j,1}\right)\Phi_{k,1}^{-1}+\sum_{l=1}^{i}H_{l,k}^{T}R_{l,k}^{-1}H_{l,k}\right]}^{-1} \end{array}$$

### 3.5. Brief Summary

The observable degree analysis method for multi-sensor information fusion system is rarely mentioned because the fusion method does not have the direct observation matrix and its observability discriminant matrix for fusion center cannot be established. The traditional observable degree analysis method is only analyzed and defined on observability, which fails when studying filtering accuracy. Thus, it is hard to reveal that there is any promotion by information fusion between fusion center and local filter before operating filtering. The observable degree of ODAEPM is defined on the main body of estimation error covariance, which is used to measure the filtering accuracy, ignoring the effect of process noise, making it possible to analyze the observable degree of fusion center in multi-sensor information fusion system.

Following these considerations, in this section, we introduce the multi-sensor information fusion problem, and list the structure of three main information fusion methods. By translating the form of estimation error covariance, we are able to get the observable degree discriminant matrix for both fusion center and local filter in each fusion method.

## 4. The Relationship of Observable Degree among Information Fusion System

The ODAEPM makes a clear relation between filtering performance and observable degree. To study how the fusion technology affects the target tracking performance, further research on the relationship of observable degree between local filter and fusion center is needed as well as on the observable degree among different fusion methods.

### 4.1. The Relationship of Observable Degree between Local Filter and Fusion Center

The distributed multi-sensor fusion system contains local filter and fusion center, and the observable degree can characterize filtering performance. By studying the relationship of observable degree between local filter and fusion center, how the information fusion affects the observation system performance can be found.

For local filter and fusion center in distributed multi-sensor fusion system without feedback, the observable degree discriminant matrix of *i*th local sensor ${\left(D_{1,k}^{*}\right)}_{i}^{dn}$ and fusion center ${\left(D_{1,k}^{*}\right)}_{f}^{dn}$ are shown in Equations (12) and (17). To find the relationship between them, a simple way is extracting the term ${\left(D_{1,k}^{*}\right)}_{i}^{dn}$ from ${\left(D_{1,k}^{*}\right)}_{f}^{dn}$. In this thought, by employing the matrix inverse lemma, we rewrite ${\left(D_{1,k}^{*}\right)}_{f}^{dn}$ as

(27) $$\begin{array}{cc} {\left(D_{1,k}^{*}\right)}_{f}^{dn} & ={\left[\Phi_{k,1}^{-T}\sum_{j=1}^{k}\sum_{l=1}^{L}\left(\Phi_{j,1}^{T}H_{l,j}^{T}R_{l,j}^{-1}H_{l,j}\Phi_{j,1}\right)\Phi_{k,1}^{-1}\right]}^{-1}\\ & ={\left[\Phi_{k,1}^{-T}\sum_{j=1}^{k}\left(\Phi_{j,1}^{T}H_{i,j}^{T}R_{i,j}^{-1}H_{i,j}\Phi_{j,1}\right)\Phi_{k,1}^{-1}+\Phi_{k,1}^{-T}\sum_{j=1}^{k}\sum_{\begin{array}{c} l=1\\ l\neq i \end{array}}^{L}\left(\Phi_{j,1}^{T}H_{l,j}^{T}R_{l,j}^{-1}H_{l,j}\Phi_{j,1}\right)\Phi_{k,1}^{-1}\right]}^{-1}\\ & ={\left(D_{1,k}^{*}\right)}_{i}^{dn}-{\left(D_{1,k}^{*}\right)}_{-i}^{dn} \end{array}$$

where

(28) $$\begin{array}{cc} {\left(D_{1,k}^{*}\right)}_{-i}^{dn}= & {\left(D_{1,k}^{*}\right)}_{i}^{dn}\Phi_{k,1}^{-T}{\left[{\left(\sum_{j=1}^{k}\sum_{\begin{array}{c} l=1\\ l\neq i \end{array}}^{L}\Phi_{j,1}^{T}H_{l,j}^{T}R_{l,j}^{-1}H_{l,j}\Phi_{j,1}\right)}^{-1}+\Phi_{k,1}^{-1}{\left(D_{1,k}^{*}\right)}_{i}^{dn}\Phi_{k,1}^{-T}\right]}^{-1}\Phi_{k,1}^{-1}{\left(D_{1,k}^{*}\right)}_{i}^{dn} \end{array}$$

while the terms ${\left(D_{1,k}^{*}\right)}_{i}^{dn}$ and $\Phi_{j,1}^{T}H_{l,j}^{T}R_{l,j}^{-1}H_{l,j}\Phi_{j,1}$ are positive semidefinite matrices, and, according to the discrimination method of positive semidefinite matrix [26], ${\left(D_{1,k}^{*}\right)}_{-i}^{dn}$ should also be a positive semidefinite matrix.

Following the rule of ODAEPM, the GOD of local sensor and fusion center are defined as:

(29) $$\begin{array}{cc} \eta_{f}^{dn} & =\frac{1}{Trace{\left(D_{1,k}^{*}\right)}_{f}^{dn}} \end{array}$$

(30) $$\begin{array}{cc} \eta_{i}^{dn} & =\frac{1}{Trace{\left(D_{1,k}^{*}\right)}_{i}^{dn}} \end{array}$$

From Equation (28) and properties of positive semi-definite matrices, we can get the following formula:

(31) $$\begin{array}{cc} Trace{\left(D_{1,k}^{*}\right)}_{f}^{dn} & =Trace[{\left(D_{1,k}^{*}\right)}_{i}^{dn}-{\left(D_{1,k}^{*}\right)}_{-i}^{dn}]\\ & =Trace{\left(D_{1,k}^{*}\right)}_{i}^{dn}-Trace{\left(D_{1,k}^{*}\right)}_{-i}^{dn} \end{array}$$

(32) $$\begin{array}{cc} Trace{\left(D_{1,k}^{*}\right)}_{-i}^{dn}\geq & 0 \end{array}$$

(33) $$\begin{array}{cc} Trace{\left(D_{1,k}^{*}\right)}_{f}^{dn}\leq & Trace{\left(D_{1,k}^{*}\right)}_{i}^{dn} \end{array}$$

Thus, it is obvious that

(34) $$\eta_{f}^{dn}\geq \eta_{i}^{dn}$$

The LOD of *j*th state component of local sensor and fusion center is defined as

(35) $$\begin{array}{cc} \Delta {\left(\eta_{f}^{dn}\right)}_{j} & =\frac{1}{{\left[{\left(D_{1,k}^{*}\right)}_{f}^{dn}\right]}_{j}} \end{array}$$

(36) $$\begin{array}{cc} \Delta {\left(\eta_{i}^{dn}\right)}_{j} & =\frac{1}{{\left[{\left(D_{1,k}^{*}\right)}_{i}^{dn}\right]}_{j}} \end{array}$$

where the subscript *j* of observable degree discriminant matrix means the *j*th diagonal element of the observable degree discriminant matrix.

To discuss the LOD, we need to extract the diagonal elements by the following equation

(37) $$\alpha_{j}={\left[\overset{j-1}{\overset{︷}{0\ldots 0}}1\ldots 0\right]}^{T}$$

Then,

(38) $$\begin{array}{cc} {\left[{\left(D_{1,k}^{*}\right)}_{f}^{dn}\right]}_{j}= & \alpha_{j}^{T}{\left(D_{1,k}^{*}\right)}_{f}^{dn}\alpha_{j} \end{array}$$

(39) $$\begin{array}{cc} D_{1,k}^{*}{{)}_{i}^{dn}]}_{j}= & \alpha_{j}^{T}{\left(D_{1,k}^{*}\right)}_{i}^{dn}\alpha_{j} \end{array}$$

(40) $$\begin{array}{cc} {\left[{\left(D_{1,k}^{*}\right)}_{i}^{dn}\right]}_{j}-{\left[{\left(D_{1,k}^{*}\right)}_{f}^{dn}\right]}_{j} & =\alpha_{j}^{T}\left[{\left(D_{1,k}^{*}\right)}_{i}^{dn}-{\left(D_{1,k}^{*}\right)}_{f}^{dn}\right]\alpha_{j}\\ & =\alpha_{j}^{T}{\left(D_{1,k}^{*}\right)}_{-i}^{dn}\alpha_{j}\geq 0 \end{array}$$

(41) $$\begin{array}{cc} D_{1,k}^{*}{{)}_{f}^{dn}]}_{j}\leq & {\left[{\left(D_{1,k}^{*}\right)}_{i}^{dn}\right]}_{j} \end{array}$$

Thus, we can get the same result of LOD between local sensor and fusion center as the GOD:

(42) $$\Delta {\left(\eta_{f}^{dn}\right)}_{j}\geq \Delta {\left(\eta_{i}^{dn}\right)}_{j}$$

Furthermore, if the local sensor in the fusion system has the same observation matrix $H_{i,j}$ for each time period, and the observation noise covariance matrix $R_{i,j}$ is also the same, it means the distributed multi-sensor fusion system consists of same kind of sensors. Under this condition, Equation (17) is rewritten as

(43) $$\begin{array}{cc} {\left(D_{1,k}^{*}\right)}_{f}^{dn} & ={\left[\Phi_{k,1}^{-T}\sum_{j=1}^{k}\sum_{i=1}^{L}\left(\Phi_{j,1}^{T}H_{i,j}^{T}R_{i,j}^{-1}H_{i,j}\Phi_{j,1}\right)\Phi_{k,1}^{-1}\right]}^{-1}\\ & =L{\left[\Phi_{k,1}^{-T}\sum_{j=1}^{k}\left(\Phi_{j,1}^{T}H_{i,j}^{T}R_{i,j}^{-1}H_{i,j}\Phi_{j,1}\right)\Phi_{k,1}^{-1}\right]}^{-1}\\ & =L{\left(D_{1,k}^{*}\right)}_{i}^{dn} \end{array}$$

which means the observation degree of fusion center in distributed multi-sensor fusion system without feedback is the sum of observation degree of its local sensors.

Next, we discuss the the relationship of observation degree between local filter and fusion center in distributed multi-sensor fusion system with feedback. According to Equations (20) and (21), we transform ${\left(D_{1,k}^{*}\right)}_{f}^{df}$ as Equation (27), making

(44) $$\begin{array}{cc} {\left(D_{1,k}^{*}\right)}_{f}^{df} & ={\left[\Phi_{k,1}^{-T}\sum_{j=1}^{k}\sum_{l=1}^{L}\left(\Phi_{j,1}^{T}H_{l,j}^{T}R_{l,j}^{-1}H_{l,j}\Phi_{j,1}\right)\Phi_{k,1}^{-1}\right]}^{-1}\\ & ={\left[\Phi_{k,1}^{-T}\sum_{j=1}^{k-1}\sum_{l=1}^{L}\left(\Phi_{j,1}^{T}H_{l,j}^{T}R_{l,j}^{-1}H_{l,j}\Phi_{j,1}\right)\Phi_{k,1}^{-1}+H_{i,k}^{T}R_{i,k}^{-1}H_{i,k}+\sum_{\begin{array}{c} l=1\\ l\neq i \end{array}}^{L}\left(H_{l,k}^{T}R_{l,k}^{-1}H_{l,k}\right)\right]}^{-1}\\ & ={\left(D_{1,k}^{*}\right)}_{i}^{df}-{\left(D_{1,k}^{*}\right)}_{-i}^{df} \end{array}$$

where

(45) $$\begin{array}{cc} {\left(D_{1,k}^{*}\right)}_{-i}^{df}= & {\left(D_{1,k}^{*}\right)}_{i}^{df}{\left[{\left(\sum_{\begin{array}{c} l=1\\ l\neq i \end{array}}^{L}H_{l,k}^{T}R_{l,k}^{-1}H_{l,k}\right)}^{-1}+{\left(D_{1,k}^{*}\right)}_{i}^{df}\right]}^{-1}{\left(D_{1,k}^{*}\right)}_{i}^{df} \end{array}$$

Following the same operation as Equations (29)–(43), we can also get the result

(46) $$\begin{array}{cc} \eta_{f}^{df} & \geq \eta_{i}^{df} \end{array}$$

(47) $$\begin{array}{cc} \Delta {\left(\eta_{f}^{df}\right)}_{j} & \geq \Delta {\left(\eta_{i}^{df}\right)}_{j} \end{array}$$

From the above proof, we come to the conclusion that, in distributed multi-sensor fusion system, whether there is feedback or non-feedback in the fusion method, both LOD and GOD of fusion center are greater than them getting the local filter. That means both in state components and system level, the fusion center obtains better filter performance than local sensor. In addition, it proves that the multi-sensor information fusion can improve the observation performance of the system from the aspect of observable degree.

### 4.2. The Relationship of Observable Degree among Different Fusion Methods

After we get the relationship between fusion center and local filter of distributed multi-sensor fusion system, we discuss the observable degree among different fusion methods.

According to Equations (17), (20) and (25), we can find the observable discrimination matrix in different fusion methods are equal to each other. Thus, we conclude that,

(48) $$\begin{array}{cc} \eta_{f}^{dn} & =\eta_{f}^{df}=\eta_{f}^{cs} \end{array}$$

(49) $$\begin{array}{cc} \Delta {\left(\eta_{f}^{dn}\right)}_{j} & =\Delta {\left(\eta_{f}^{df}\right)}_{j}=\Delta {\left(\eta_{f}^{cs}\right)}_{j} \end{array}$$

the LOD and GOD of fusion center in these fusion methods are the same. Then, we discuss the observable degree of local filter in distributed multi-sensor fusion system with or without feedback and the observable degree of different steps during sequential fusion.

First, we compare the observable degree of local filter in distributed multi-sensor fusion system with or without feedback. Doing the same as Equation (27), ${\left(D_{1,k}^{*}\right)}_{i}^{df}$ is rewritten as

(50) $$\begin{array}{cc} {\left(D_{1,k}^{*}\right)}_{i}^{df} & ={\left[\Phi_{k,1}^{-T}\sum_{j=1}^{k-1}\sum_{l=1}^{L}\left(\Phi_{j,1}^{T}H_{l,j}^{T}R_{l,j}^{-1}H_{l,j}\Phi_{j,1}\right)\Phi_{k,1}^{-1}+H_{i,k}^{T}R_{i,k}^{-1}H_{i,k}\right]}^{-1}\\ & ={\left[\Phi_{k,1}^{-T}\left(\sum_{j=1}^{k}\Phi_{j,1}^{T}H_{i,j}^{T}R_{i,j}^{-1}H_{i,j}\Phi_{j,1}\right)\Phi_{k,1}^{-1}+\Phi_{k,1}^{-T}\sum_{j=1}^{k-1}\sum_{\begin{array}{c} l=1\\ l\neq i \end{array}}^{L}\left(\Phi_{j,1}^{T}H_{l,j}^{T}R_{l,j}^{-1}H_{l,j}\Phi_{j,1}\right)\Phi_{k,1}^{-1}\right]}^{-1}\\ & ={\left(D_{1,k}^{*}\right)}_{i}^{dn}-{\left(D_{1,k}^{*}\right)}_{-dn}^{df} \end{array}$$

where

(51) $$\begin{array}{cc} {\left(D_{1,k}^{*}\right)}_{-dn}^{df}= & {\left(D_{1,k}^{*}\right)}_{i}^{dn}\Phi_{k,1}^{-T}{\left[{\left(\sum_{j=1}^{k-1}\sum_{\begin{array}{c} l=1\\ l\neq i \end{array}}^{L}\Phi_{j,1}^{T}H_{l,j}^{T}R_{l,j}^{-1}H_{l,j}\Phi_{j,1}\right)}^{-1}+\Phi_{k,1}^{-1}{\left(D_{1,k}^{*}\right)}_{i}^{dn}\Phi_{k,1}^{-T}\right]}^{-1}\Phi_{k,1}^{-1}{\left(D_{1,k}^{*}\right)}_{i}^{dn} \end{array}$$

Following the same operation as Equations (29)–(43), the conclusion is

(52) $$\begin{array}{cc} \eta_{i}^{df} & \geq \eta_{i}^{dn} \end{array}$$

(53) $$\begin{array}{cc} \Delta {\left(\eta_{i}^{df}\right)}_{j} & \geq \Delta {\left(\eta_{i}^{dn}\right)}_{j} \end{array}$$

Secondly, compare the observable degree between local filter in distributed multi-sensor fusion system with feedback and different steps during sequential fusion. Consider that the ranking of sequential input of sequential fusion system is in ascending sort order of index of the sensor number. Following the same operation as above,

(54) $$\begin{array}{cc} {\left(D_{1,k}^{*}\right)}_{i}^{cs} & ={\left[\Phi_{k,1}^{-T}\sum_{j=1}^{k-1}\sum_{l=1}^{L}\left(\Phi_{j,1}^{T}H_{l,j}^{T}R_{l,j}^{-1}H_{l,j}\Phi_{j,1}\right)\Phi_{k,1}^{-1}+\sum_{l=1}^{i}H_{l,k}^{T}R_{l,k}^{-1}H_{l,k}\right]}^{-1}\\ & ={\left[\Phi_{k,1}^{-T}\sum_{j=1}^{k-1}\sum_{l=1}^{L}\left(\Phi_{j,1}^{T}H_{l,j}^{T}R_{l,j}^{-1}H_{l,j}\Phi_{j,1}\right)\Phi_{k,1}^{-1}+H_{i,k}^{T}R_{i,k}^{-1}H_{i,k}+\sum_{l=1}^{i-1}\left(H_{l,j}^{T}R_{l,j}^{-1}H_{l,j}\right)\right]}^{-1}\\ & ={\left(D_{1,k}^{*}\right)}_{i}^{df}-{\left(D_{1,k}^{*}\right)}_{-df}^{cs} \end{array}$$

where

(55) $$\begin{array}{cc} {\left(D_{1,k}^{*}\right)}_{-df}^{cs}= & {\left(D_{1,k}^{*}\right)}_{i}^{df}[{\left(\sum_{l=1}^{i-1}{\left(H_{l,j}^{T}R_{l,j}^{-1}H_{l,j}\right)}^{-1}+{\left(D_{1,k}^{*}\right)}_{i}^{df}\right]}^{-1}{\left(D_{1,k}^{*}\right)}_{i}^{df} \end{array}$$

Thus, the result is

(56) $$\begin{array}{cc} \eta_{i}^{cs} & \geq \eta_{i}^{df} \end{array}$$

(57) $$\begin{array}{cc} \Delta {\left(\eta_{i}^{cs}\right)}_{j} & \geq \Delta {\left(\eta_{i}^{df}\right)}_{j} \end{array}$$

The relationship of observable degree among the local filter in distributed multi-sensor fusion system with or without feedback and different step during sequential fusion is concluded as follows

(58) $$\begin{array}{cc} \eta_{i}^{dn} & \leq \eta_{i}^{df}\leq \eta_{i}^{cs} \end{array}$$

(59) $$\begin{array}{cc} \Delta {\left(\eta_{i}^{dn}\right)}_{j} & \leq \Delta {\left(\eta_{i}^{df}\right)}_{j}\leq \Delta {\left(\eta_{i}^{cs}\right)}_{j} \end{array}$$

### 4.3. Brief Summary

According to [15], the observability definition of ODAEPM, the greater the observability, the better the filtering performance lead us to the the following conclusions:

In two different distributed multi-sensor fusion systems, both GOD and LOD of the fusion center are better than the local filters.That means both in state components and system level the fusion center obtains better filter performance than local sensor. In addition, it proves that the multi-sensor information fusion can improve the observation performance of the system from the aspect of observable degree.

In two different distributed multi-sensor fusion systems, the feedback information from the fusion center can improve the filtering performance of the local filter, but with or without feedback information, the fusion solution at the fusion center is equivalent. That means both in state components and system level the local filter of distributed multi-sensor fusion systems with feedback obtains better filter performance than the local filter of distributed multi-sensor fusion systems without feedback.

From the perspective of observability, in centralized multi-sensor fusion systems, the fusion solution at the fusion center is equivalent to the others. The filtering performance of the fusion center is also improved at *i*th step during time *k*.This improvement is superior to the distributed multi-sensor with feedback system for the improvement of local filter filtering performance.

## 5. Numerical Analysis

To validate the effectiveness of the mathematical proof of relationship of observable degree among information fusion system, computer simulation was performed to demonstrate the target tracking with three different observation sensors. The sensors used in this paper are only used to obtain the speed and displacement of CV models, so we only use the velocity sensor and the displacement sensor. The target motion is the typical two-dimensional linear discrete motion models as constant velocity (CV) model.

The state transition matrix is given as:

$$\Phi =\left[\begin{array}{cccc} 1 & t & 0 & 0\\ 0 & 1 & 0 & 0\\ 0 & 0 & 1 & t\\ 0 & 0 & 0 & 1 \end{array}\right]$$

The observation matrix of three different sensors are:

$$\begin{array}{c} H_{1}=\left[\begin{array}{cccc} 1 & 0 & 0 & 0\\ 0 & 0 & 1 & 0 \end{array}\right],H_{2}=\left[\begin{array}{cccc} 1 & 1 & 0 & 0\\ 0 & 0 & 1 & 1 \end{array}\right],H_{3}=\left[\begin{array}{cccc} 0 & 1 & 0 & 0\\ 0 & 0 & 0 & 1 \end{array}\right] \end{array}$$

The observation covariance matrix of them are:

$$R_{1}=\left[\begin{array}{cc} 0.1 & 0\\ 0 & 0.1 \end{array}\right],R_{2}=\left[\begin{array}{cc} 0.2 & 0\\ 0 & 0.2 \end{array}\right],R_{3}=\left[\begin{array}{cc} 0.3 & 0\\ 0 & 0.3 \end{array}\right]$$

The subscripts of **H** and **R** correspond to same index sensor.

**Figure 5:**
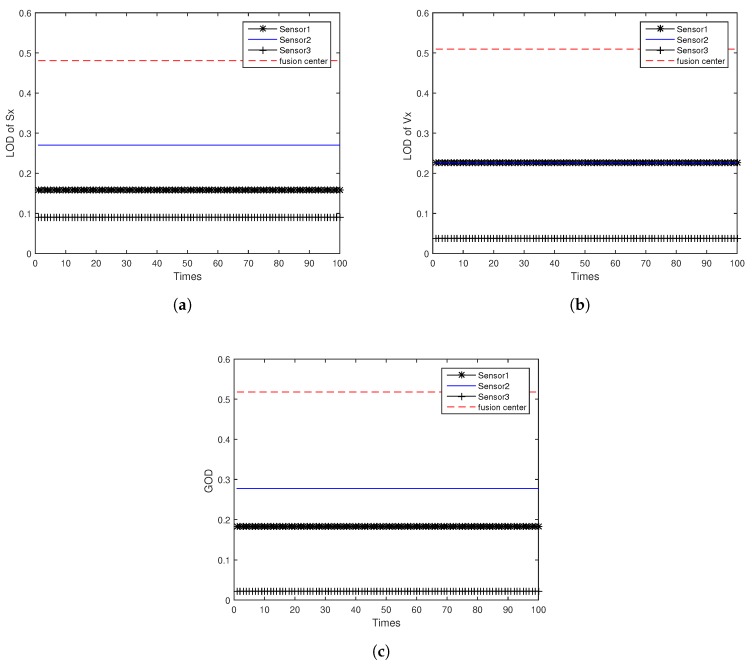
Observable degree comparison between local filter and fusion center in distributed multi-sensor fusion system without feedback: (**a**) LOD of *Sx*; (**b**) LOD of *Vx*; (**c**) GOD.

**Figure 6:**
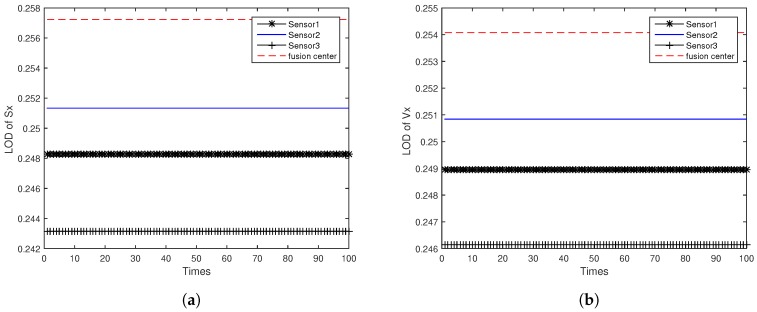

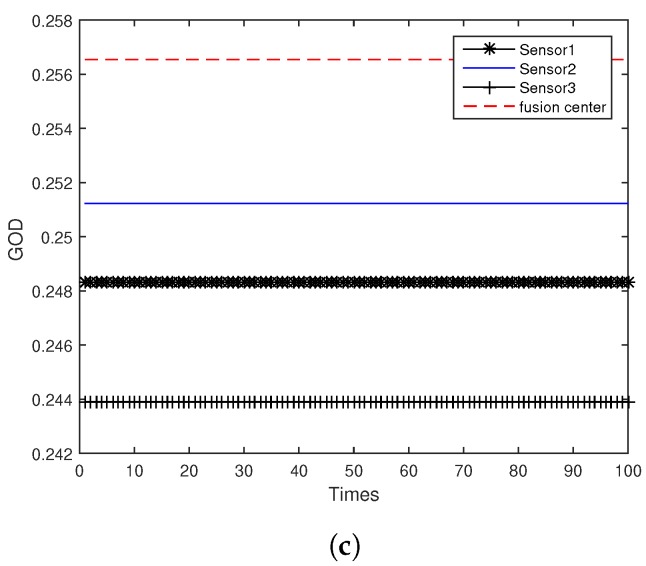
Observable degree comparison between local filter and fusion center in distributed multi-sensor fusion system with feedback: (**a**) LOD of *Sx*; (**b**) LOD of *Vx*; (**c**) GOD.

**Figure 7:**
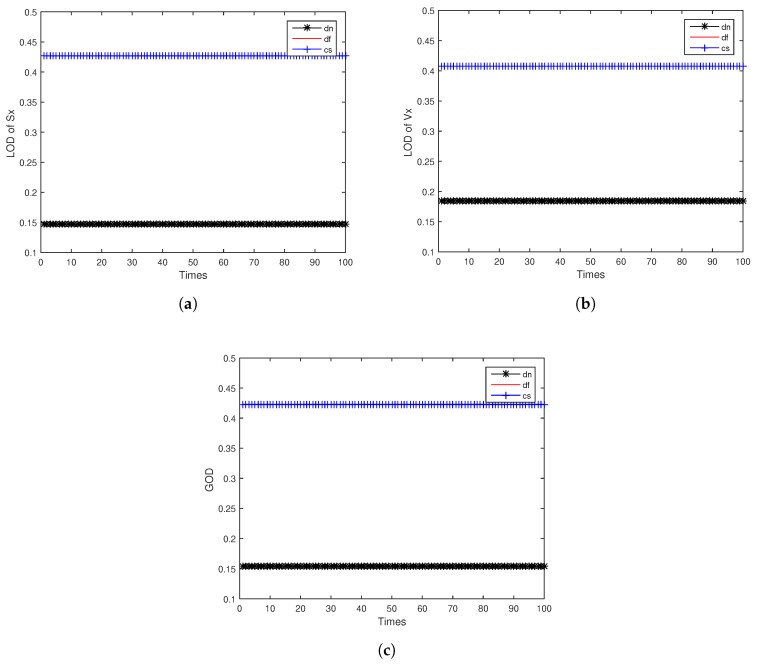
Observable degree comparison among different fusion methods for same index 1: (**a**) LOD of *Sx*; (**b**) LOD of *Vx*; (**c**) GOD.

**Figure 8:**
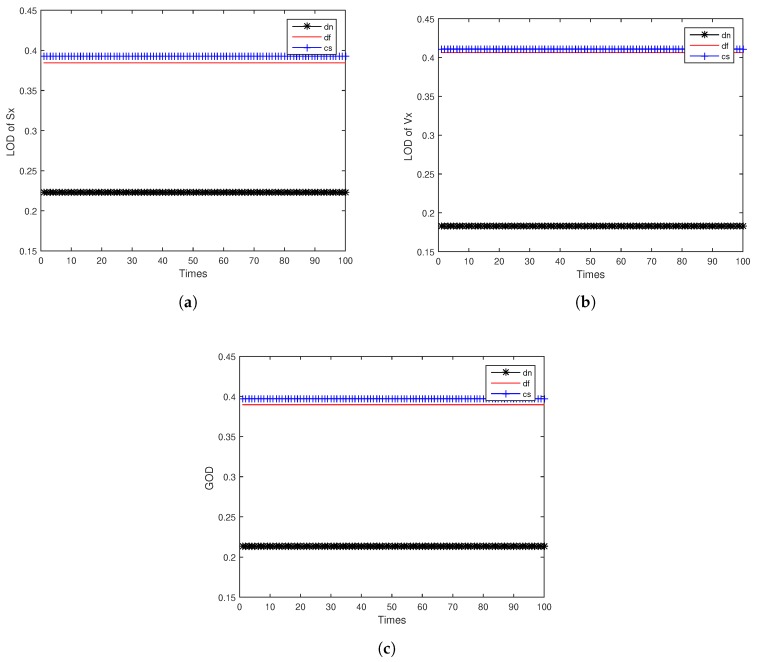
Observable degree comparison among different fusion methods for same index 2: (**a**) LOD of *Sx*; (**b**) LOD of *Vx*; (**c**) GOD.

**Figure 9:**
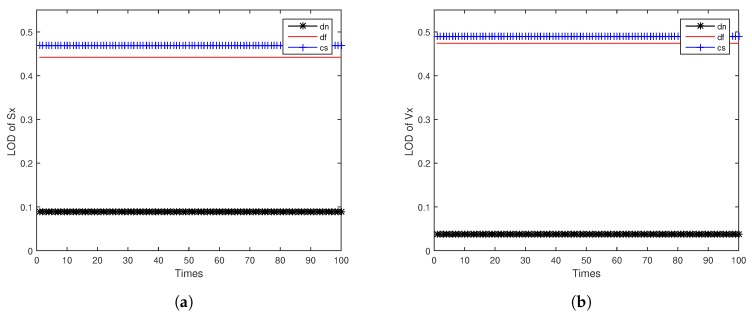

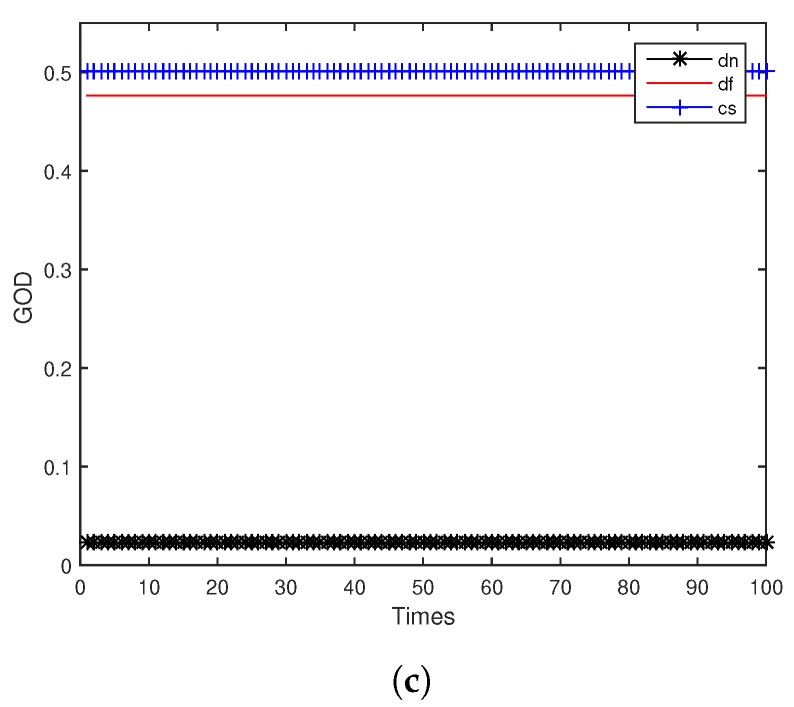
Observable degree comparison among different fusion methods for same index 3:(**a**) LOD of *Sx*; (**b**) LOD of *Vx*; (**c**) GOD.

The simulation results are shown in Figure 5, Figure 6, Figure 7, Figure 8 and Figure 9.

Furthermore, to make the relationship clearer, the observable degree analysis in the normalized form, as

(60) $$\eta^{\prime }=\frac{\eta }{\sum \eta }$$

where the numerator is the observable degree obtained by ODAEPM and the denominator is the sum of all observable degrees participating in the comparison in the same figure.

Figure 5a,b shows the LOD of target position Sx and velocity Vx for ODAEPM obtained in distributed multi-sensor fusion system without feedback, both observable degree of three local filters and fusion center are contained. The GODs of local filter and fusion center using distributed multi-sensor fusion method without feedback are shown in Figure 5c. Similar to Figure 5, Figure 6 shows the condition of LOD and GOD of local filter and fusion center of distributed multi-sensor fusion system with feedback. In Figure 5 and Figure 6, we can find that both LOD and GOD obtained from fusion center are greater than those obtained from local filter, whether the fusion method returning the fusion result to local filter as a feedback. It confirms that the information fusion can obtain better performance than single sensor observation.

Figure 7 shows the observable degree comparison among different fusion methods for same index 1, while the index 1 for two distributed multi-sensor fusion method means the observable degree obtained from the first local sensor, and for centralized multi-sensor sequential fusion system that means it is the first step of sequential fusion at this period, the fusion method only grabs the observation data from the first local sensor. Similarly, Figure 8 and Figure 9 show the observable degree comparison among different fusion methods for same index 2 and 3, for centralized multi-sensor sequential fusion system increased acquisition the information for the two other local sensors. For each figure, both LOD and GOD are well compared. With the assumption that the sequence of sequential fusion system input is the same as the sensor index, the simulation results in Figure 7, Figure 8 and Figure 9 show that the obtained observable degree in ascending order is the distributed multi-sensor fusion system without feedback, the distributed multi-sensor fusion system with feedback, and the centralized multi-sensor sequential fusion system. It provides the correctness of the derivation for relationship among different fusion methods in this paper. The obtained observable degree will be the same when obtained from distributed multi-sensor fusion system with feedback and centralized multi-sensor sequential fusion system only under the index equal to 1.

**Figure 10:**
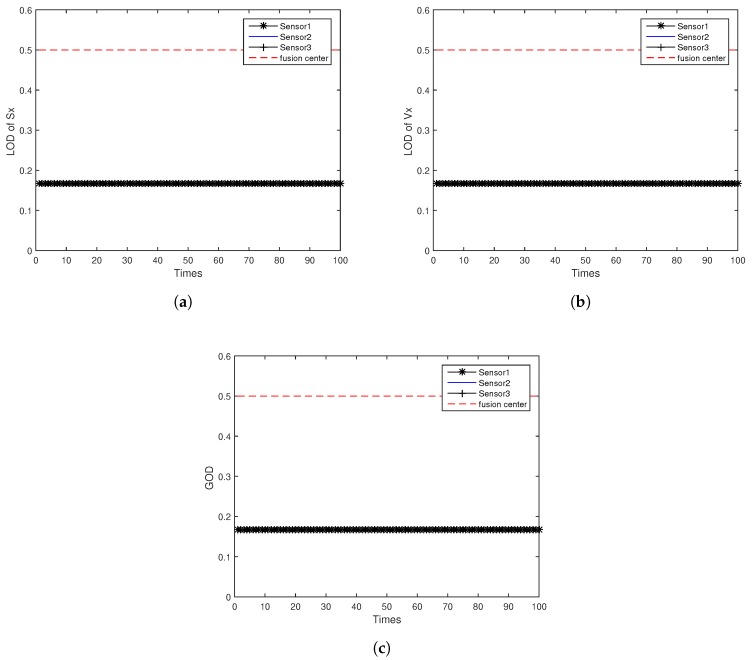
Observable degree of local filter and fusion center:(**a**) LOD of *Sx*; (**b**) LOD of *Vx*; (**c**) GOD.

Then, to prove the result shown in Equation (44), if the distributed multi-sensor fusion system consists of the same kind of sensors, the observation degree of fusion center in distributed multi-sensor fusion system without feedback is the sum of observation degree of its local sensors. We established the multi-sensor fusion system without feedback with three sensors, the observation matrix and observation noise covariance matrix of which are $H_{1}$ and $R_{1}$. The simulation is shown in Table 1 and Figure 10.

In Table 1 and Figure 10, we can see the observable degree of fusion center is equal to the sum of its local sensors.

Based on the simulation results presented in Figure 5, Figure 6, Figure 7, Figure 8, Figure 9, Figure 10 and Table 1, our proof of observable degree relationship among multi-sensor fusion system is verified.

## 6. Conclusions

In this paper, observable degree analysis for multi sensor fusion system is addressed. Three different multi-sensor fusion system methods were studied, and the observability of three different fusion methods was calculated according to the definition of ODAEPM. It is also proved mathematically that, in two different distributed multi-sensor fusion systems, both GOD and LOD of their respective fusion centers are higher than their local filter, that is, the filtering performance is also better, which also illustrates the advantages of multi-sensor fusion system. The local filter performance of the distributed multi-sensor fusion system with feedback is better than without feedback. This shows that the feedback information from the fusion center can effectively improve the performance of the local filter. To confirm the mathematical proof, we simulated three different kinds of fusion methods operating on two-dimensional linear discrete motion constant velocity models and established three different observation sensors. We can verify our mathematical conclusions based on the simulation. It is clear from simulation that the feedback mechanism for distributed multi-sensor systems can significantly improve the performance of local filter. Therefore, in a multi-sensor system, the feedback mechanism should be introduced to the local nodes as much as possible, which can improve the filtering performance of the local filter. 

## Figures and Tables

**Table 1:** Observable degree of local filter and fusion center with condition that fusion system consists of the same kind of sensors.

	Sensor1	Sensor2	Sensor3	Fusion Center
*Sx*	253.8	253.8	253.8	761.3
*Vx*	8832.5	8832.5	8832.5	26,497.5
system	246.3	246.3	246.3	738.8
